# Three new *Penicillium* species (section *Lanata-Divaricata*, *Aspergillaceae*, *Eurotiales*) from agricultural soil in Yunnan, China

**DOI:** 10.3897/mycokeys.138.198488

**Published:** 2026-08-14

**Authors:** Xiankun Zhang, Shuailiang Shi, Zhongwen Duan, Wenqi Lai, Zefen Yu

**Affiliations:** 1 State Key Laboratory for Conservation and Utilization of Bio-Resources in Yunnan, Yunnan University, Kunming 650504, China School of Life Sciences, Yunnan University Kunming China; 2 Yunnan Key Laboratory of Basic Research and Innovative Application for Green Biological Production, Yunnan University, Kunming 650504, China State Key Laboratory for Conservation and Utilization of Bio-Resources in Yunnan, Yunnan University Kunming China; 3 School of Life Sciences, Yunnan University, Kunming 650504, China Yunnan Key Laboratory of Basic Research and Innovative Application for Green Biological Production, Yunnan University Kunming China

**Keywords:** *Lanata-Divaricata*, multi-locus phylogeny, new species, taxonomy

## Abstract

Eight fungal strains isolated from agricultural soils in mountainous regions of Yunnan Province, China, were identified as three novel species of *Penicillium* section *Lanata-Divaricata* based on morphological characteristics and multi-locus phylogenetic analyses. Phylogenetic inference using four gene regions, including the internal transcribed spacer (ITS) region, β-tubulin (*BenA*), calmodulin (*CaM*), and the second largest subunit of RNA polymerase II (*RPB2*), supported the recognition of these isolates as distinct species. The new taxa are described here as *P. longyangense* (series *Janthinella*), *P. longlingense* (series *Dalearum*), and *P. zhenyuanense* (series *Rolfsiorum*). Morphological comparisons with closely related species further confirmed their taxonomic placement. The discovery of these species expands the known diversity of *Penicillium* section *Lanata-Divaricata* and improves our understanding of the species distribution of soilborne *Penicillium* in mountainous agricultural ecosystems of Yunnan, China.

## Introduction

*Eurotiales* is a species-rich order comprising several taxonomically and economically important genera, such as *Aspergillus* P. Micheli ex Haller, *Penicillium* Link, and *Talaromyces* C.R. Benj. (Houbraken et al. 2020; Visagie et al. 2024). Species identification in these genera has historically been challenging due to morphological convergence and limited diagnostic characters (Peterson 2006; Visagie et al. 2024). Recently, integrative taxonomy combining morphological characters and multilocus phylogenetic analyses has improved species delimitation and classification in *Penicillium* and related genera, resulting in a more stable taxonomic framework (Visagie et al. 2014, 2016a; Houbraken et al. 2020).

The genus *Penicillium* is one of the most geographically widespread and species-rich genera of filamentous fungi and is commonly isolated from soil and other environments (Park et al. 2019; Hao et al. 2020). In recent years, more than 500 species of *Penicillium* have been formally described worldwide (Houbraken et al. 2020). The genus is divided into two subgenera, *Aspergilloides* and *Penicillium*, further subdivided into sections and series (Houbraken et al. 2020).

Section *Lanata-Divaricata* (belonging to subgenus *Aspergilloides*) was introduced by Thom (1930) and later reinstated based on molecular phylogenetic evidence (Houbraken and Samson 2011; Houbraken et al. 2020). This section is subdivided into five series (*Dalearum*, *Janthinella*, *Oxalica*, *Rolfsiorum*, and *Simplicissima*) and comprises more than 90 species (Houbraken et al. 2020; Visagie et al. 2024). Members of this section are characterised by divaricate conidiophores and colonies with broadly spreading margins, although phenotypic characters may overlap among species (Houbraken and Samson 2011; Visagie et al. 2014; Houbraken et al. 2020). Therefore, multilocus phylogenetic analyses, especially using ITS, *BenA*, *CaM*, and *RPB2*, or combined datasets of ITS, *BenA*, *CaM*, and *RPB2*, have been widely used for species delimitation and phylogenetic placement in this section (Visagie et al. 2014; Diao et al. 2019; Houbraken et al. 2020; Visagie et al. 2024).

Isolates of this section have been obtained from a broad range of substrates—including soil, pollen, and endophytic plant tissues—with soil being the dominant habitat (Houbraken and Samson 2011; Visagie et al. 2016b; Barbosa et al. 2018; Diao et al. 2019; Pangging et al. 2021; Torres-Garcia et al. 2022). Several species of *Penicillium* section *Lanata-Divaricata* have been reported from acidic, humus-rich or organic matter-rich substrates, particularly soils and decaying plant materials (Johnson 1998; Gross and Robbins 2000; Dhakar et al. 2014; Visagie et al. 2015; Diao et al. 2019). Yunnan Province is characterised by complex mountainous and plateau topography, where pronounced vertical zonation of soil types generates strong environmental heterogeneity in pH, organic matter content, and nutrient availability (Ming and Tang 2016; Xiao et al. 2021). Such heterogeneous forest ecosystems, enriched with organic matter inputs, provide diverse microhabitats that may facilitate the ecological differentiation and diversification of soilborne *Penicillium* lineages (Song et al. 2024).

During a survey of *Penicillium* diversity in agricultural soils from mountainous regions of Yunnan Province, China (including Baoshan and Pu’er cities), eight isolates were obtained and found to represent three previously undescribed species. Multi-locus phylogenetic analyses based on ITS, *BenA*, *CaM*, and *RPB2*, combined with detailed morphological observations, supported their placement in *Penicillium* section *Lanata-Divaricata*. These species are here described as *Penicillium longyangense* (series *Janthinella*), *Penicillium longlingense* (series *Dalearum*), and *Penicillium zhenyuanense* (series *Rolfsiorum*). This study expands the known diversity of section *Lanata-Divaricata* and provides new insights into the taxonomy, ecology, and geographic distribution of soilborne *Penicillium* species in Yunnan Province, China.

## Material and methods

### Sampling and isolation

Rhizosphere soil samples were collected from agricultural fields in Pu’er and Baoshan cities, Yunnan Province, China. From each site, ten subsamples were collected from the 5–10 cm soil layer and combined into one composite sample. The composite samples were placed in sterile bags, transported to the laboratory, and stored at 4 °C prior to processing within one week.

For fungal isolation, 10 g of each soil sample was suspended in 90 mL sterile distilled water and shaken at 180 rpm for 30 min. The suspension was allowed to settle for 5 min, and 1 mL of the supernatant was serially diluted tenfold. Aliquots (100 μL) of 10^−2^ and 10^−3^ dilutions were spread onto Rose Bengal agar (RBA) supplemented with penicillin and streptomycin (50 μg/mL each) to inhibit bacterial growth (Martin 1950; Aldossari and Ishii 2021). Plates were incubated at 28 °C for 3–5 days. Emerging colonies were transferred to potato dextrose agar (PDA) and purified by repeated subculturing. A total of 32 *Penicillium* isolates were obtained, of which eight were selected for further taxonomic analyses.

Pure cultures were deposited in the Herbarium of the Laboratory for Conservation and Utilization of Bio-Resources, Yunnan University (**YMF**), Kunming, China, and the ex-type cultures were additionally deposited in the Guangdong Microbial Culture Collection Center (**GDMCC**), Institute of Microbiology, Guangdong Academy of Sciences, Guangzhou, China.

### DNA extraction, amplification and sequencing

Genomic DNA was extracted from fresh mycelia following Ye et al. (2023) with minor modifications. Approximately 0.5 g of mycelium was scraped from PDA cultures and transferred to sterile tubes containing a steel bead. A total of 800 μL urea extraction buffer (7 M urea, 50 mM Tris-HCl, 1% SDS, 150 mM NaCl; pH 8.0) was added, and samples were homogenised for 5 min. The homogenate was centrifuged at 12,000 rpm for 5 min and the supernatant was collected. An equal volume of phenol–chloroform–isoamyl alcohol (25:24:1) was added and centrifuged under the same conditions. DNA was precipitated using an equal volume of isopropanol and one-tenth volume of 3 M sodium acetate, followed by incubation at –20 °C for 20 min. The DNA pellet was washed with 70% ethanol, air-dried, resuspended in 50 μL ddH_2_O, and stored at –20 °C.

Four loci were amplified, including the internal transcribed spacer (ITS), β-tubulin (*BenA*), calmodulin (*CaM*), and RNA polymerase II second largest subunit (*RPB2*). Primer pairs ITS5/ITS4, Bt2a/Bt2b, CF1/Cmd6, and rpb2-5F2/rpb2-7R were used for ITS, *BenA*, *CaM*, and *RPB2*, respectively (White et al. 1990; Glass and Donaldson 1995; Hong et al. 2006; Sung et al. 2007; Peterson 2008). PCR reactions were performed in 25 μL volumes containing 1 μL genomic DNA, 1 μL of each primer (10 μM), 12.5 μL 2× MasterMix (Tiangen Biotech, Beijing, China), and 9.5 μL ddH2O. Amplifications were conducted using an Eppendorf Mastercycler (Eppendorf, Hamburg, Germany) following Visagie et al. (2014). PCR products were purified and sequenced in both directions using an ABI 3730XL DNA sequencer (Applied Biosystems, Foster City, CA, USA). Newly generated sequences were deposited in GenBank (https://www.ncbi.nlm.nih.gov/genbank/) and accession numbers are listed in Table 1.

**Table 1.** Strains and GenBank accession numbers used in the phylogenetic analyses of *Penicillium*.

**Species**	**Strain**	**Substrate/Host and locality**	**GenBank accession number**			
			**ITS**	***BenA***	***CaM***	***RPB2***
*P. abidjanum*	CBS 246.67^T^	Savannah soil, Ivory Coast	GU981582	GU981650	MN969234	JN121469
*P. amphipolaria*	DAOMC 250551^T^	Soil, Quartermain Mountains, Antarctic Dry Valleys, Antarctica	KT887872	KT887833	KT887794	MN969177
*P. annulatum*	CBS 135126^T^	Air, Stellenbosch, South Africa	JX091426	JX091514	JX141545	KF296410
*P. ausonanum*	FMR 16948^T^	Fluvial sediment, Spain	LR655808	LR655809	LR655810	LR655811
*P. austrosinense*	CBS 144505^T^	Acidic soil, Hainan, China	KY495007	KY495116	MN969328	KY495061
*P. bissettii*	CBS 140972^T^	*Picea* forest soil, Québec, Canada	KT887845	KT887806	KT887767	MN969178
*P. boreae*	CBS 111717^T^	Unknown source, Unknown country	AF481122	JN617715	AF481138	MN969107
*P. brefeldianum*	CBS 235.81^T^	Human alimentary tract, Unknown country	AF033435	GU981623	EU021683	KF296421
*P. camponoti*	CBS 140982^T^	Carpenter ant (*Camponotus pennsylvanicus*), New Brunswick, Canada	KT887855	KT887816	KT887777	MN969179
*P. caperatum*	CBS 443.75^T^	Soil, NSW, Australia	KC411761	GU981660	MN969242	KF296422
*P. coeruleum*	CBS 141.45^T^	Unknown source, Unknown country	GU981606	GU981655	MN969247	KF296425
*P. coffeatum*	CGMCC 3.25152^T^	Soil, China	OQ870815	OR051121	OR051298	OR051466
*P. cremeogriseum*	CBS 223.66^T^	Forest soil, Kiev, Ukraine	GU981586	GU981624	MN969250	KF296426
*P. curticaule*	CBS 135127^T^	Soil, Malmesbury, South Africa	FJ231021	JX091526	JX141536	KF296417
*P. daleae*	CBS 211.28^T^	Conifer soil, Poland	GU981583	GU981649	MN969251	KF296427
*P. donggangicum*	AS 3.15900^T^	Tidal-flat soil, Liaoning, China	MW946996	MZ004914	MZ004918	MW979253
*P. ehrlichii*	CBS 324.48^T^	Unknown substrate, Poland	GU981578	GU981652	MN969253	KF296428
*P. elleniae*	CBS 118135^T^	Forest leaf litter, Araracuara, Colombia	GU981612	GU981663	MN969254	KF296429
*P. excelsum*	DTO 357-D7^T^	*Bertholletia excelsa* nut shell, Amazon, Brazil	KR815341	KP691061	KR815342	MN969166
*P. flaviroseum*	CBS 144479^T^	Acidic soil, Hainan, China	KY495032	KY495141	MN969329	KY495083
*P. fructuariae-cellae*	CBS 145110^T^	Corvina withered grapes, Verona, Italy	MK039434	KU554679	MK045337	MK520927
*P. glaucoroseum*	CBS 138908^T^	Soil, Virginia, USA	MN431390	MN969383	MN969257	MN969119
*P. griseopurpureum*	CBS 406.65^T^	*Pinus* soil, Lancashire, UK	KF296408	KF296467	MN969261	KF296431
*P. guaibinense*	CCDCA 11512^T^	Soil, China	MH674389	MH674391	MH674393	–
*P. hainanense*	CBS 144527^T^	Acidic soil, Hainan, China	KY495009	KY495118	MN969333	KY495062
*P. janthinellum*	CBS 340.48^T^	Soil, Nicaragua	GU981585	GU981625	MN969268	JN121497
*P. javanicum*	CBS 341.48^T^	*Camellia sinensis* root, Java, Indonesia	GU981613	GU981657	MN969269	JN121498
*P. jianfenglingense*	CBS 144640^T^	Acidic soil, Hainan, China	KY495016	KY495125	MN969334	KY495069
*P. koreense*	KACC 47721^T^	Soil, South Korea	KJ801939	KM000846	MN969317	MN969159
*P. laevigatum*	NN 072364^T^	Acidic soil, Hainan, China	KY495015	KY495124	MN969335	KY495068
*P. limosum*	CBS 339.97^T^	Marine sediment, Nagasaki, Japan	GU981568	GU981621	MN969271	KF296433
*P. lineolatum*	CBS 188.77^T^	Copse soil, Japan	GU981579	GU981620	MN969272	KF296434
*P. ludwigii*	CBS 417.68^T^	Polished *Oryza sativa* seed, Japan	KF296409	KF296468	MN969273	KF296435
***P. longlingense***	**YMF 1.11137^T^**	**Soil, Yunnan, China**	**PZ279979**	**PZ306492**	**PZ306501**	**PZ306510**
***P. longlingense***	**YMF 1.11153**	**Soil, Yunnan, China**	**PZ279981**	**PZ306494**	**PZ306503**	**PZ306512**
***P. longlingense***	**YMF 1.11156**	**Soil, Yunnan, China**	**PZ279980**	**PZ306493**	**PZ306502**	**PZ306511**
***P. longlingense***	**YMF 1.11658**	**Soil, Yunnan, China**	**PZ344665**	**PZ347309**	**PZ357595**	**PZ330332**
***P. longyangense***	**YMF 1.10573^T^**	**Soil, Yunnan, China**	**PZ279982**	**PZ306495**	**PZ306504**	**PZ306513**
***P. longyangense***	**YMF 1.11657**	**Soil, Yunnan, China**	**PZ344664**	**PZ347308**	**PZ357594**	**PZ330331**
*P. malacosphaerulum*	CBS 135120^T^	Soil, Malmesbury, South Africa	FJ231026	JX091524	JX141542	KF296438
*P. melanosporum*	CBS 146938^T^	Soil, Spain	LR655192	LR655196	LR655200	LR655204
*P. meloforme*	CBS 445.74^T^	Soil, Papua New Guinea	KC411762	GU981656	MN969276	KF296440
*P. michoacanense*	FMR 17612^T^	Soil, Mexico	LR655194	LR655198	LR655202	LR655206
*P. nordestinense*	URM 8423^T^	Pollen from *Melipona scutellaris* nests, Brazil	OV265270	OV265324	OV265272	OM927721
*P. ochrochloron*	CBS 357.48^T^	Copper sulphate solution, Washington, USA	GU981604	GU981672	MN969280	KF296445
*P. ortum*	CBS 135669^T^	Forest soil, Kiev, Ukraine	JX091427	JX091520	JX141551	KF296443
*P. penarojense*	CBS 113178^T^	Forest leaf litter, Peña Roja, Colombia	GU981570	GU981646	MN969287	KF296450
*P. piscarium*	CBS 362.48^T^	Cod-liver oil emulsion, Norway	GU981600	GU981668	MN969288	KF296451
*P. pulvillorum*	CBS 280.39^T^	Acidic soil, UK	AF178517	GU981670	MN969289	KF296452
*P. pulvillorum*	CBS 275.83	Rye grain, Spain	GU981601	GU981671	KC346336	KF296423
*P. raperi*	CBS 281.58^T^	Soil, Bedford, UK	AF033433	GU981622	MN969291	KF296453
*P. reticulisporum*	CBS 122.68^T^	Soil, Japan	AF033437	MN969394	MN969293	KF296454
*P. rolfsii*	CBS 368.48^T^	Unknown source, Unknown country	JN617705	GU981667	MN969294	KF296455
*P. rotoruae*	CBS 145838^T^	*Pinus radiata* timber, New Zealand	MN315103	MN315104	MN315102	MT240842
*P. rubriannulatum*	NN 072456^T^	Acidic soil, Hainan, China	KY495029	KY495138	MN969336	KY495080
*P. siccitolerans*	FMR 17381^T^	Soil, Spain	LR655193	LR655197	LR655201	LR655205
*P. singorense*	CBS 138214^T^	House dust, Songkhla, Thailand	KJ775674	KJ775167	KJ775403	MN969138
*P. soli*	KUMCC 18-0202^T^	Soil, China	MT152337	MT161681	MT178249	MT384372
*P. soliforme*	CBS 144482^T^	Acidic soil, Hainan, China	KY495038	KY495147	MN969337	KY495047
*P. stangiae*	URM 8347^T^	Soil, Brazil	MW648590	MW646388	MW646390	MW646392
*P. subrubescens*	CBS 132785^T^	*Helianthus tuberosus* soil, Helsinki, Finland	KC346350	KC346327	KC346330	KC346306
*P. svalbardense*	CBS 122416^T^	Glacial ice, Svalbard, Norway	GU981603	DQ486644	KC346338	KF296457
*P. terrarumae*	CBS 131811^T^	Heavy-metal-contaminated soil, Guizhou, China	MN431397	KX650295	MN969323	MN969185
*P. tibetense*	CGMCC 3.28597^T^	Rhizosphere soil, Tibet, China	PQ643284	PQ519857	PQ519858	PQ519859
*P. vanderhammenii*	CBS 126216^T^	Forest leaf litter, Araracuara, Colombia	GU981574	GU981647	MN969308	KF296458
*P. vasconiae*	CBS 339.79^T^	Acid-washed brown soil, Spain	GU981599	GU981653	MN969309	MN969144
*P. viridissimum*	CBS 144484^T^	Acidic soil, Hainan, China	KY495004	KY495113	MN969339	KY495059
*P. yunnanense*	CBS 144485^T^	Acidic soil, Yunnan, China	KY494990	KY495099	MN969340	KY495048
***P. zhenyuanense***	**YMF 1.11149^T^**	**Soil, Yunnan, China**	**PZ344666**	**PZ347310**	**PZ357596**	**PZ330333**
***P. zhenyuanense***	**YMF 1.11656**	**Soil, Yunnan, China**	**PZ344667**	**PZ347311**	**PZ357597**	**PZ330334**
*P. zonatum*	CBS 992.72^T^	Coastal marsh soil, North Carolina, USA	GU981581	GU981651	MN969315	KF296461

Note: T = Ex-type; New isolates are in bold; The line “–” represents the absence of GenBank record.

### Phylogenetic analysis

Sequences of ITS, *BenA*, *CaM*, and *RPB2* obtained in this study were initially compared with reference sequences in GenBank using BLAST to determine their closely related species. Reference strains were selected from recent taxonomic studies of *Penicillium* section *Lanata-Divaricata*. A total of 70 ingroup strains representing 64 species of series *Janthinella*, *Dalearum* and *Rolfsiorum* were included. *Penicillium boreae* S.W. Peterson & Sigler CBS 111717^T^ (section *Stolkia*) was chosen as the outgroup.

Sequences for each locus were aligned using ClustalX v1.83 and manually edited in BioEdit v7.0. The individual alignments were concatenated into a combined dataset, and ambiguously aligned regions were excluded. Maximum likelihood (ML) analysis was conducted using IQ-TREE 2 (Minh et al. 2020), with the concatenated dataset partitioned by locus and the best-fit substitution models and partitioning scheme selected using ModelFinder under the MFP+MERGE option (Kalyaanamoorthy et al. 2017). Branch support was assessed using 1,000 ultrafast bootstrap replicates. Bayesian inference (BI) analysis was performed in MrBayes v3.1.2 under the GTR+G substitution model (Ronquist and Huelsenbeck 2003). Four independent runs with four Markov chains were executed for 5,000,000 generations, sampling every 500 generations. The first 25% of trees were discarded as burn-in. The average standard deviation of split frequencies fell below 0.01, indicating convergence among the independent runs. Nodes with bootstrap support (BS) ≥ 75% and posterior probabilities (PP) ≥ 0.90 were considered statistically supported. The final phylogeny was inferred from the ML analysis and visualized using TreeView.

### Morphological characterisation

For morphological observation, a small amount of conidial suspension or mycelial fragments was taken from the margin of actively growing colonies on potato dextrose agar (PDA) using a sterile inoculation needle. Point inoculations were performed at three equidistant points (forming an equilateral triangle) on the surface of the following media: malt extract agar (MEA), Czapek yeast autolysate agar (CYA), and yeast extract-sucrose agar (YES). Cultures were incubated in the dark at 25 °C for 7 days. Colony characteristics, colony diameters, and pigment production were recorded for each strain on MEA, CYA, and YES.

Slide cultures were prepared following Cai et al. (2009) to observe micromorphological structures. Microscopic features were observed using an Olympus BX51 microscope (Tokyo, Japan) equipped with differential interference contrast (DIC) optics. Photomicrographs were captured using an Olympus DP10 digital camera operated with Olympus DP Controller software (Version 3.1.1208). Thirty individual structures were randomly measured, and average morphological dimensions were calculated.

## Results

### Phylogenetic analyses

BLAST searches based on ITS, *BenA*, *CaM*, and *RPB2* sequences indicated that the eight isolates belonged to *Penicillium* section *Lanata-Divaricata*. Species-level sequence similarities based on the ex-type isolates of the three proposed species are summarized in Table 2.

**Table 2.** Pairwise sequence similarities of ITS, *BenA*, *CaM* and *RPB2* between the ex-type strains of the three *Penicillium* species described in this study and their closest relatives in section *Lanata-Divaricata*.

**Species**	**Ex-type isolate**	**Closest species**	**Reference /type strain**	**Series**	**ITS (%)**	***BenA* (%)**	***CaM* (%)**	***RPB2* (%)**
*P. longyangense*	YMF 1.10573^T^	*P. koreense*	KACC 47721^T^	*Janthinella*	99.01	98.98	98.94	98.52
*P. longlingense*	YMF 1.11137^T^	*P. daleae*	CBS 211.28^T^	*Dalearum*	99.22	99.75	98.24	99.34
*P. zhenyuanense*	YMF 1.11149^T^	*P. bissettii*	CBS 140972^T^	*Rolfsiorum*	97.55	97.45	97.49	96.65

Note: Sequence similarity values were calculated using the ex-type strains of the three proposed species and their closest known relatives. All eight isolates examined in this study are listed in Table 1 and were included in the combined multilocus phylogenetic analysis.

**Figure 1.**
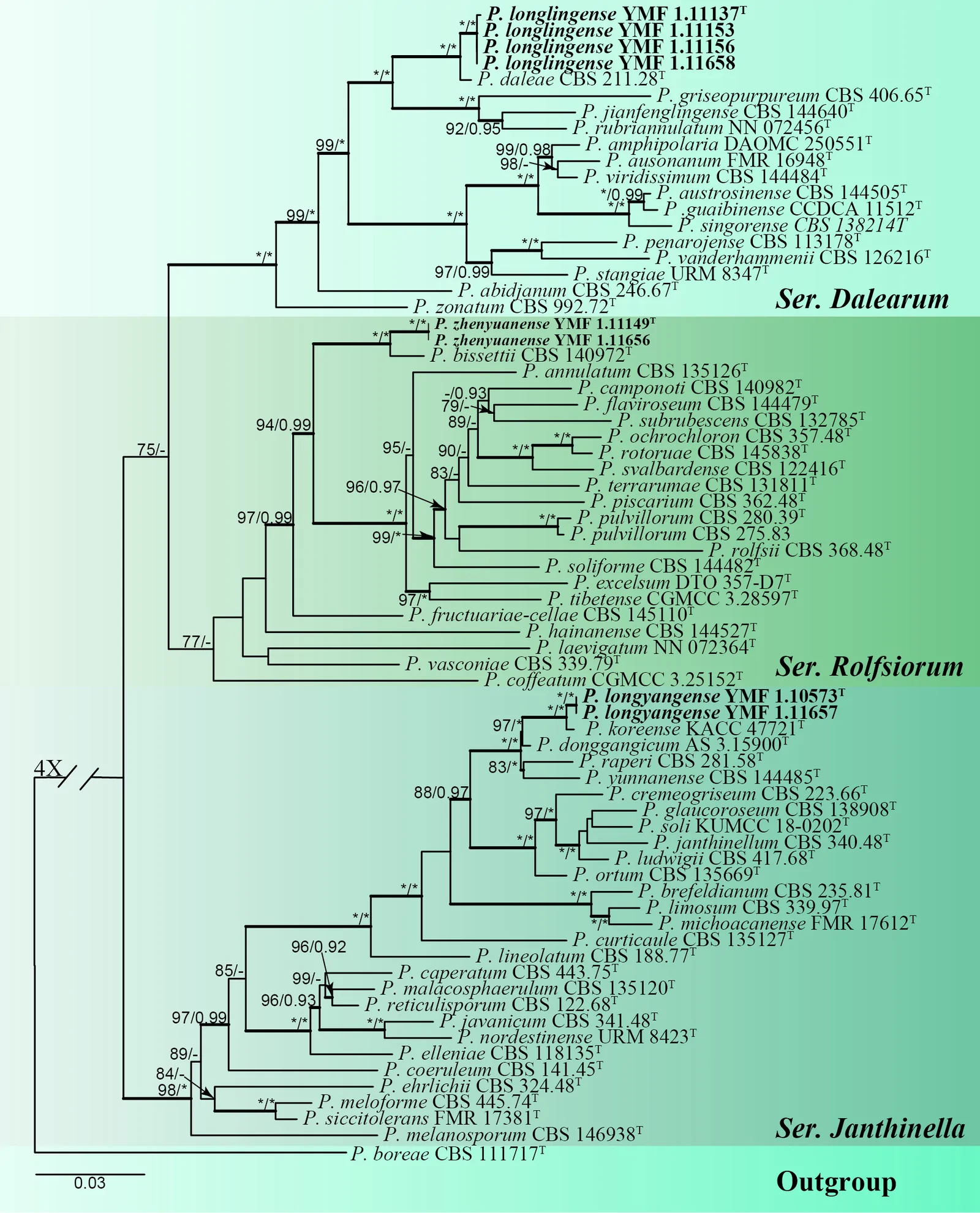
Phylogenetic tree of *Penicillium* species based on the combined ITS, *BenA*, *CaM* and *RPB2* sequences, inferred using maximum likelihood. Bootstrap values (BS ≥ 75%) and Bayesian posterior probabilities (PP ≥ 0.90) are shown at the nodes. Branches with BS ≥ 75% and PP ≥ 0.90 are shown in bold. The branch marked “4 ×” was shortened to one-quarter of its original length for presentation. *Penicillium boreae* CBS 111717^T^ was used as the outgroup. Newly described species are indicated in bold. An asterisk indicates BS = 100% or PP = 1.00; ^T^ indicates ex-type strain.

To clarify their phylogenetic positions and species boundaries, a concatenated dataset of ITS, *BenA*, *CaM*, and *RPB2* sequences was constructed, including 71 strains representing three series (*Janthinella*, *Dalearum*, and *Rolfsiorum*). The final alignment comprised 2,188 characters (ITS: 476 bp, *BenA*: 435 bp, *CaM*: 517 bp, and *RPB2*: 760 bp). Maximum likelihood and Bayesian inference produced congruent tree topologies with strong support values (Fig. 1). The phylogenetic tree assigned the eight isolates to three proposed species placed in three different series of section *Lanata-Divaricata*: (1) *P. longyangense* was placed in series *Janthinella* and formed a sister lineage to *P. koreense* S.B. Hong, D.H. Kim & Y.H. You (100% BS, 1.00 PP); (2) *P. longlingense* was assigned to series *Dalearum* and clustered with *P. daleae* K.W. Zaleski (100% BS, 1.00 PP); (3) *P. zhenyuanense* was recovered in series *Rolfsiorum* and grouped with *P. bissettii* Visagie & Seifert (100% BS, 1.00 PP).

### Taxonomy

#### Penicillium longyangense

Taxon classificationFungiEurotialesAspergillaceae

Z. F. Yu & X. K. Zhang sp. nov.

C89D8F97-58B7-5DBC-B920-481B552DE125

863617

Fig. 2

##### Etymology.

The epithet refers to Longyang District, the type locality.

**Figure 2.**
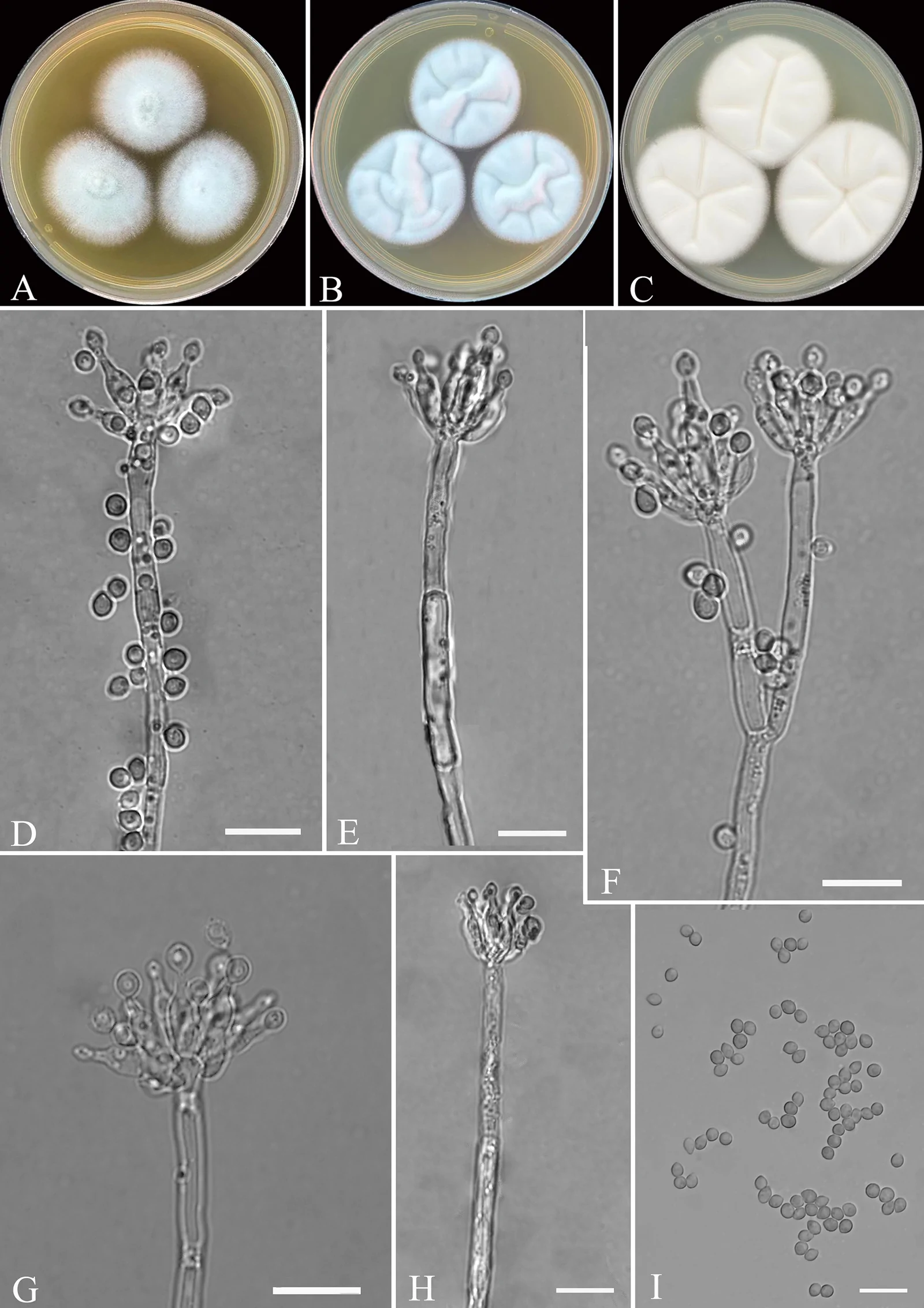
Colonies and microscopic characteristics of *Penicillium longyangense*YMF 1.10573. **A–C**. Colony obverse on MEA, YES and CYA after 7 d at 25 °C in darkness; **D–H**. Conidiophores and phialides; **I**. Conidia. Scale bars: 10 μm.

##### Type.

China • Yunnan Province, Baoshan City, Longyang District, Lujiangba. 24°56'34.8"N, 98°50'44.5"E, 916.7 m alt., isolated from agricultural soil in a kidney bean (*Phaseolus vulgaris*) field, Dec. 2024, Z.F. Yu & X.K. Zhang (holotype YMFD 1.10573, dried culture; ex-type culture YMF 1.10573 = GDMCC 3.1370).

##### Subgeneric classification.

Subgenus *Aspergilloides*, section *Lanata-Divaricata*, series *Janthinella*.

##### Description.

Conidiophores smooth-walled, thin-walled, 170–570 × 2–3 μm (mean = 288 × 2.5 μm, n = 30). ***Penicilli*** monoverticillate, compact, terminal, occasionally with a divaricate branch. ***Metulae*** absent. ***Phialides*** ampulliform, borne in terminal whorls of 8–12, 6.5–11.5 × 2–3.5 μm (mean = 8.7 × 2.8 μm, n = 30). ***Conidia*** globose to subglobose, smooth-walled, 2–3 μm diam. (mean = 2.8 μm, n = 30).

##### Colony diam.

(in mm) 7 days, 25 °C: MEA 19–21, YES 22–24, CYA 24–26.

##### Culture characteristics.

7 days, 25 °C: Colonies on MEA flat, white to pale yellowish, floccose at the margin, dominated by aerial hyphae, sporulation sparse, pale green at the centre, exudate and soluble pigments absent. Colonies on YES floccose, centrally raised, radially sulcate, white, margin distinct, regular, sporulation scarce, exudate and soluble pigments absent. Colonies on CYA dense, floccose to fluffy, wrinkled, radially sulcate, centrally depressed, white to pale buff; margin regular, slightly fimbriate, sporulation sparse, exudate and soluble pigments absent. Colour descriptions follow Rayner (1970).

##### Additional materials examined.

China • Yunnan Province, Baoshan City, Longyang District, Lujiangba; isolated from agricultural soil in a kidney bean (*Phaseolus vulgaris*) field; Dec. 2024; Z.F. Yu; living culture YMF 1.11657.

##### Notes.

Phylogenetically, *P. longyangense* is closely related to *P. koreense* within the series *Janthinella* (Fig. 1). It differs from *P. koreense* by its slower growth on CYA and MEA after 7 d at 25 °C (24–26 mm and 19–21 mm, respectively, vs. 32–34 mm and 42–48 mm); paler colonies with sparse to scarce sporulation and absence of exudates and soluble pigments, whereas *P. koreense* produces grey-green conidia en masse, shows moderate to strong sporulation, and produces clear exudate droplets on CYA; shorter conidiophores (170–570 × 2–3 μm vs. 200–800 × 2–3 μm), generally narrower phialides (6.5–11.5 × 2–3.5 μm vs. 8–11.5 × 3–4 μm), and globose to subglobose conidia (2–3 μm diam. vs. 2.5–3.5 × 2–3 μm, globose to broadly ellipsoidal) (You et al. 2014). These phylogenetic and morphological differences support the recognition of *P. longyangense* as a distinct species.

#### Penicillium longlingense

Taxon classificationFungiEurotialesAspergillaceae

Z. F. Yu & X. K. Zhang sp. nov.

95C6DE3C-6FEF-5ADF-B7B8-F2E96A6D79D1

863618

Fig. 3

##### Etymology.

The epithet refers to Longling County, the type locality.

**Figure 3.**
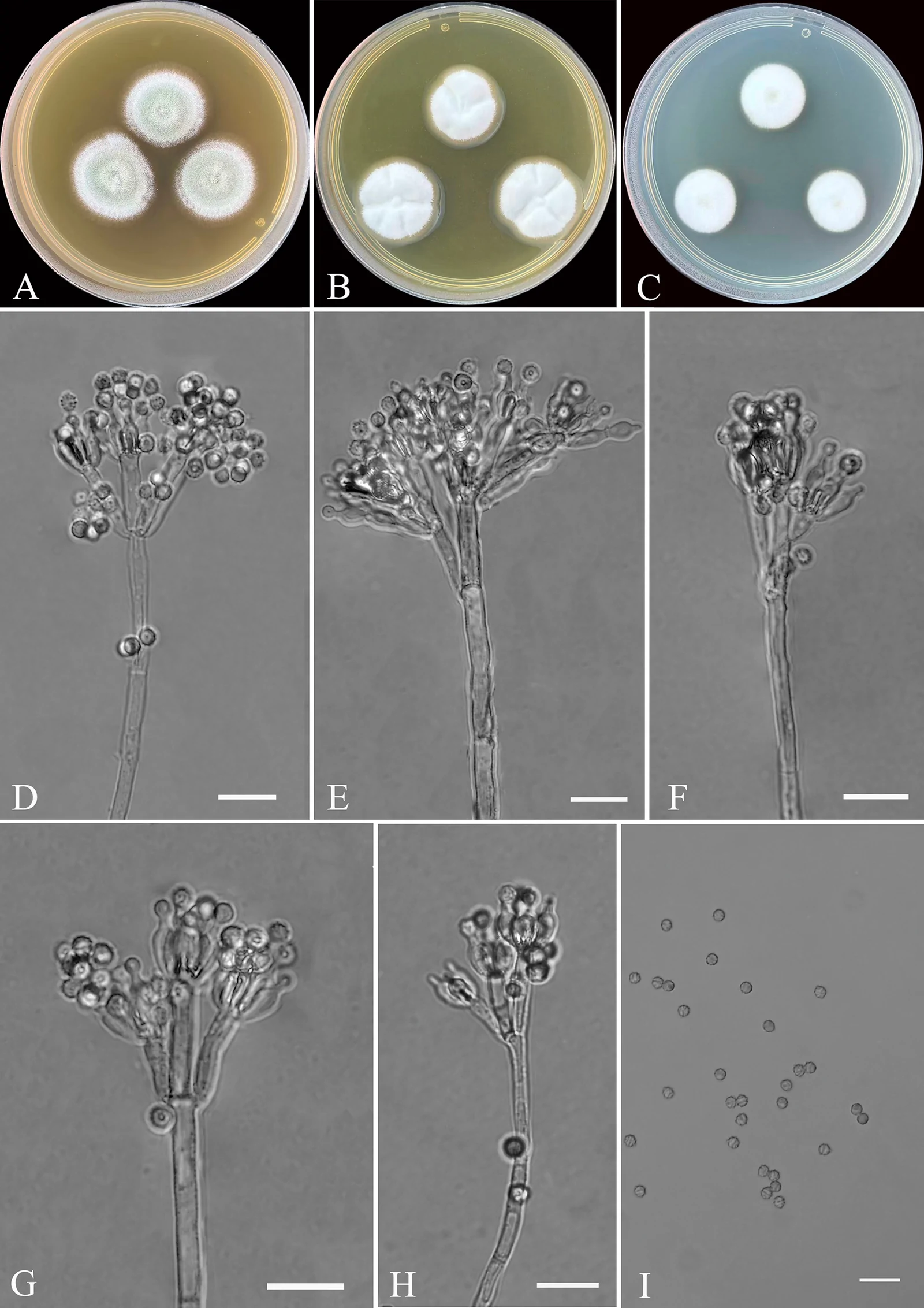
Colonies and microscopic characteristics of *Penicillium longlingense*YMF 1.11137. **A–C**. Colony obverse on MEA, YES and CYA after 7 d at 25 °C in darkness; **D–H**. Conidiophores and phialides; **I**. Conidia. Scale bars: 10 μm.

##### Type.

China • Yunnan Province, Baoshan City, Longling County, Longjiang. 24°49'12"N, 98°44'34"E, 1,919.47 m alt., isolated from soil in a sweet potato (*Ipomoea batatas*) field, Sep. 2025, Z.F. Yu & X.K. Zhang (holotype YMFD 1.11137, dried culture; ex-type culture YMF 1.11137 = GDMCC 3.1371).

##### Subgeneric classification.

Subgenus *Aspergilloides*, section *Lanata-Divaricata*, series *Dalearum*.

##### Description.

***Conidiophores*** smooth-walled, thin-walled, hyaline, mostly unbranched, occasionally furcate or branched near the apex, 80–190 × 2.5–4 μm (mean = 127 × 3 μm, n = 30). ***Penicilli*** biverticillate, compact to irregularly branched. ***Metulae*** divergent to appressed, 10–20 × 2–3.5 μm (mean = 15 × 2.9 μm, n = 30). ***Phialides*** ampulliform, borne in whorls of 4–6, 5–9 × 2–3.5 μm, with inconspicuous collarettes (mean = 6.9 × 2.6 μm, n = 30). Conidia globose to subglobose, finely roughened, 2–4 μm diam. (mean = 2.8 μm, n = 30).

##### Colony diam.

(in mm) 7 days, 25 °C: MEA 16–18, YES 15–17, CYA 14–16.

##### Culture characteristics.

7 days, 25 °C: Colonies on MEA are floccose to lanose, sporulation is prolific in the centre, coloured leek green, sporulation density decreases progressively from the centre to the periphery, the colour becoming paler, and a well-defined white margin develops at the extreme edge, exudate and soluble pigments absent. Colonies on YES low to slightly raised, white, weakly radially sulcate to wrinkled; margin well defined and regular, sporulation sparse to nearly absent, exudate and soluble pigments absent. Colonies on CYA white, dense, floccose, low to slightly raised, margin regular, sporulation sparse, exudate and soluble pigments absent. Colour descriptions follow Rayner (1970).

##### Additional materials examined.

China • Yunnan Province, Baoshan City, Longling County, Longjiang; isolated from soil in a sweet potato (*Ipomoea batatas*) field; Sep. 2025; Z.F. Yu; living cultures YMF 1.11153, YMF 1.11156, YMF 1.11658.

##### Notes.

Phylogenetically, *P. longlingense* is closely related to *P. daleae* (Fig. 1), but differs in several diagnostic cultural and morphological characters. In culture, *P. longlingense* grows more slowly on CYA and MEA after 7 d at 25 °C (14–16 mm and 16–18 mm, respectively) than *P. daleae* (30–34 mm and 25–35 mm, respectively) and lacks exudates and soluble pigments on both media; by contrast, *P. daleae* may produce clear brown exudates on CYA and typically produces yellow to reddish brown soluble pigments on MEA. Micromorphologically, *P. longlingense* has generally wider and slightly longer conidiophores (80–190 × 2.5–4 μm vs. 35–170 × 2.3–2.7 μm). Phialide dimensions partially overlap, although *P. longlingense* generally produces shorter and less variable phialides (5–9 × 2–3.5 μm vs. 6–7(–12) × 2.4–3.0 μm in *P. daleae*). In addition, *P. longlingense* produces globose to subglobose, finely roughened conidia (2–4 μm diam., mean = 2.8 μm), whereas *P. daleae* has spheroidal, conspicuously spinose conidia (2.6–3.2 × (2–)2.5–3 μm) (Cho et al. 2005). These differences distinguish *P. longlingense* from *P. daleae* and support its recognition as a distinct species in series *Dalearum*.

#### Penicillium zhenyuanense

Taxon classificationFungiEurotialesAspergillaceae

Z. F. Yu & X. K. Zhang sp. nov.

32018D5F-E0FF-577D-802D-21B87E6347EF

863619

Fig. 4

##### Etymology.

The specific epithet refers to Zhenyuan County, the type locality of this species.

**Figure 4.**
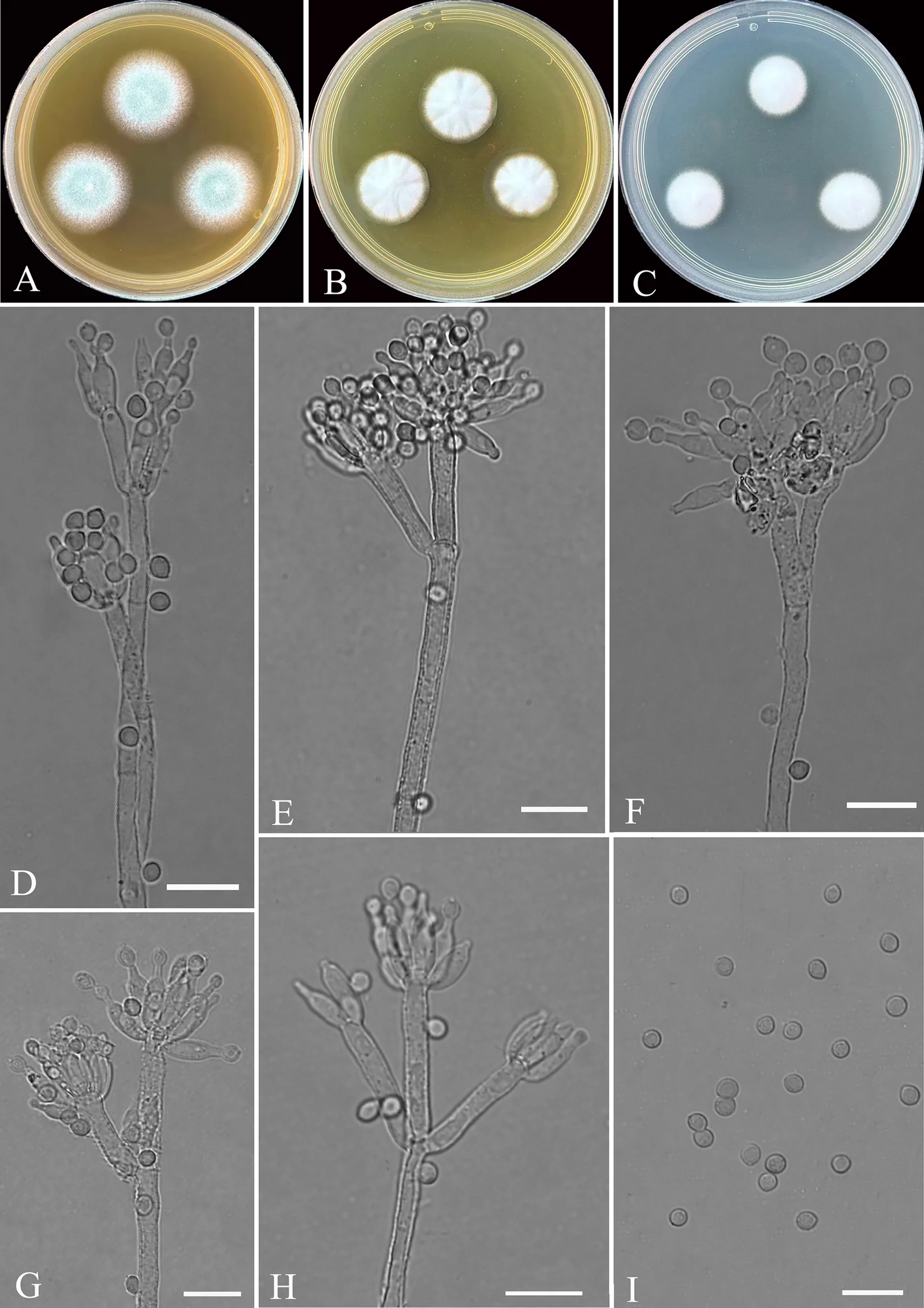
Colonies and microscopic characteristics of *Penicillium zhenyuanense*YMF 1.11149. **A–C**. Colony obverse on MEA, YES and CYA after 7 d at 25 °C in darkness; **D–H**. Conidiophores and phialides; **I**. Conidia. Scale bars: 10 μm.

##### Type.

China • Yunnan Province, Pu’er City, Zhenyuan Yi, Hani and Lahu Autonomous County, isolated from soil in a tea (*Camellia sinensis*) plantation, 23°56'29.8"N, 101°29'54.4"E, 2363.7 m alt, Sep. 2025, Z.F. Yu & X.K. Zhang (holotype YMFD 1.11149, dried culture; ex-type culture YMF 1.11149 = GDMCC 3.1372).

##### Subgeneric classification.

Subgenus *Aspergilloides*, section *Lanata-Divaricata*, series *Rolfsiorum*.

##### Description.

***Conidiophores*** smooth-walled, thin-walled, 80–300 × 2–4 μm (mean = 147 × 3 μm, n = 30), simple or occasionally branched near the apex. ***Penicilli*** mostly monoverticillate, occasionally with short apical or subapical branches, appearing reduced biverticillate, compact to irregularly branched. ***Metulae***, when present, short, poorly differentiated. ***Phialides*** ampulliform to slightly lageniform, borne in terminal whorls or on short apical and subapical branches, usually 4–9 per whorl, 7–12 × 1.5–3.5 μm (mean = 9.1 × 2.6 μm, n = 30). ***Conidia*** globose to subglobose, smooth to finely roughened, 2.5–3.5 μm diam. (mean = 2.9 μm, n = 30).

##### Colony diam.

(in mm) 7 days, 25 °C: MEA 20–22, YES 13–15, CYA 15–17.

##### Culture characteristics.

7 days, 25 °C: Colonies on MEA floccose to lanose, with dense sporulation at the centre, grey-green, becoming paler towards the margin, margin well defined, white, exudates and soluble pigments absent. Colonies on YES low to slightly raised, white, weakly radially sulcate to wrinkled, margin well defined, regular, sporulation sparse to nearly absent, exudate and soluble pigments absent. Colonies on CYA dense, floccose, white to pale buff, low to slightly raised, weakly radially sulcate, margin regular, sporulation sparse, exudate and soluble pigments absent. Colour descriptions follow Rayner (1970).

##### Additional materials examined.

China • Yunnan Province, Pu’er City, Zhenyuan Yi, Hani and Lahu Autonomous County; isolated from soil in a tea (*Camellia sinensis*) field; Sep. 2025; Z.F. Yu; living culture YMF 1.11656.

##### Notes.

Phylogenetically, *P. zhenyuanense* is closely related to *P. bissettii* within series *Rolfsiorum* (Fig. 1), but differs from *P. bissettii* by its markedly slower growth after 7 d at 25 °C on CYA, MEA and YES (15–17, 20–22 and 13–15 mm diam., respectively, vs. 42–43 mm on CYA, 35–43 mm on MEA and 47–52 mm on YES). In addition, *P. zhenyuanense* forms paler colonies, with grey-green sporulation mainly restricted to the colony centre on MEA and sparse to nearly absent sporulation on CYA and YES, whereas *P. bissettii* produces more rapidly spreading colonies with moderately dense sporulation on CYA and YES, pinkish red submerged mycelium on MEA, and brown to dark brown or brownish orange reverses on CYA and YES. Micromorphologically, *P. zhenyuanense* differs from *P. bissettii* in having smooth-walled, shorter conidiophores (80–300 × 2–4 μm vs. rough-walled stipes, 190–670 × 2.5–3.5 μm), mostly monoverticillate to occasionally reduced biverticillate penicilli, and short, poorly differentiated metulae when present, rather than more complex biverticillate to terverticillate penicilli with well-developed metulae. Conidia of *P. zhenyuanense* are globose to subglobose, smooth to finely roughened and slightly larger on average (2.5–3.5 μm diam.) than those of *P. bissettii* (2–3 μm diam.) (Visagie et al. 2016b). Together, these phylogenetic, cultural and morphological differences support the recognition of *P. zhenyuanense* as a distinct species in series *Rolfsiorum*.

## Discussion

The present study describes three novel species of *Penicillium* section *Lanata-Divaricata*—*P. longyangense*, *P. longlingense*, and *P. zhenyuanense*—from agricultural soils in mountainous areas of Yunnan Province, China. Section *Lanata-Divaricata* is a species-rich lineage within *Penicillium* subgenus *Aspergilloides*. Recent taxonomic revisions have refined the circumscription of this section and established a series-level infrasectional framework based primarily on multilocus phylogenetic relationships (Houbraken et al. 2020; Visagie et al. 2024). Species delimitation in this group generally requires an integrative approach because morphological characters may overlap among closely related taxa. Accordingly, the taxonomic conclusions of this study were based on congruent evidence from multilocus phylogenetic analyses and cultural and micromorphological characters.

Southwestern China, particularly Yunnan and adjacent areas, represents a hotspot for the discovery of new *Penicillium* species, yet Yunnan itself remains substantially undersampled, indicating that considerable *Penicillium* diversity remains to be uncovered in its soil ecosystems (Rong et al. 2016; Diao et al. 2019; Doilom et al. 2020; Xu et al. 2022; Wang et al. 2023; Song et al. 2024; Liang et al. 2024, 2025). Against this regional background, the three taxa described here help fill an important gap in knowledge of the diversity of section Lanata-Divaricata in Yunnan and warrant rigorous evaluation of their taxonomic distinctness.

To assess this taxonomic distinctness, multilocus phylogenetic analyses based on the ITS, *BenA*, *CaM* and *RPB2* loci provided robust topological support for delimiting the eight isolates as three phylogenetically distinct species. Because ITS often provides limited resolution among closely related *Penicillium* species, protein-coding loci such as *BenA*, *CaM* and *RPB2* are commonly used in combination, and species boundaries are not generally defined by a fixed level of sequence divergence at a single locus (Schoch et al. 2012; Visagie et al. 2014; Houbraken et al. 2020). In the present study, sequence similarities between the proposed species and their closest relatives remained high at some individual loci, ranging from 96.65% to 99.75% across the four loci (Table 2). Nevertheless, the concatenated four-locus dataset resolved all eight isolates into three independent monophyletic lineages, each represented by at least two isolates and receiving full support under both maximum likelihood and Bayesian inference (MLBS/BPP = 100%/1.00; Fig. 1). *Penicillium longyangense*, *P. longlingense* and *P. zhenyuanense* were clearly separated from their respective closest relatives, *P. koreense*, *P. daleae* and *P. bissettii*, and all three sister-group relationships were also fully supported (MLBS/BPP = 100%/1.00; Fig. 1). These phylogenetic relationships were congruent with diagnostic cultural and micromorphological differences. *Penicillium longyangense* differs from *P. koreense* KACC 47721^T^ mainly in its slower growth on CYA and MEA, paler and more sparsely sporulating colonies, and generally shorter conidiophores and narrower phialides. *P. longlingense* differs from *P. daleae* CBS 211.28^T^ principally in its slower growth on CYA and MEA, absence of exudates and soluble pigments, and globose to subglobose conidia. *P. zhenyuanense* differs from *P. bissettii* CBS 140972^T^ mainly in its markedly slower growth, sparse sporulation, predominantly monoverticillate penicilli and shorter, smooth-walled conidiophores. Therefore, the recognition of *P. longyangense*, *P. longlingense* and *P. zhenyuanense* is not based on sequence divergence percentages alone, but on combined evidence from multilocus phylogenetic placement, representation by multiple isolates and diagnostic phenotypic differences. This taxonomic strategy is consistent with recent systematic studies of section *Lanata-Divaricata*, in which morphological characters and multilocus molecular data are jointly used as the principal criteria for species delimitation (Xu et al. 2022; Nóbrega et al. 2024; Liang et al. 2025; Supapongsri et al. 2025).

Species of *Penicillium* section *Lanata-Divaricata* have been recovered from diverse habitats, including acidic soils, coastal tidal flats, freshwater sediments, cave substrates, mangrove ecosystems, plant rhizospheres and bulk soils (Diao et al. 2019; Xu et al. 2022; Torres-Garcia et al. 2022; Nóbrega et al. 2024; Liang et al. 2025; Supapongsri et al. 2025). In the present study, the three novel taxa were isolated from agricultural soils in Baoshan and Pu’er, Yunnan Province. Although regional surveys have reported acidic or variable soil pH conditions in Longling, Longyang and Pu’er (Baoshan Agriculture and Rural Affairs Bureau 2021; Hu et al. 2022; Zhou et al. 2024), these regional data do not represent direct measurements at the sampling sites. Because soil pH, organic matter content, nutrient availability and other physicochemical properties were not measured *in situ*, any proposed associations of the new species with acidic or organic matter-rich soils, as well as any inferences regarding niche differentiation, remain speculative and require verification.

Some *Penicillium* strains have been associated with nutrient mobilization, plant–fungus interactions, tolerance to acidic or metal-stressed soil conditions, and the production of diverse secondary metabolites (Doilom et al. 2020; El Hajj Assaf et al. 2020; Suraby et al. 2023; Dhandapani et al. 2024). However, no functional assays were conducted for the three new species in the present study. Consequently, their potential roles in phosphate solubilisation, acid tolerance, secondary metabolite production and plant-associated interactions remain unverified. Future studies should expand sampling across cultivated, semi-natural and natural habitats, integrate site-specific analyses of soil pH, organic matter content, nutrient availability, moisture and texture, and conduct controlled functional assays, including assays of pH tolerance, phosphate solubilization and plant–fungus interactions, together with extrolite profiling. These approaches will help clarify the habitat distributions, environmental adaptability and potential ecological functions of *P. longyangense*, *P. longlingense* and *P. zhenyuanense*.

In conclusion, *P. longyangense*, *P. longlingense* and *P. zhenyuanense* are formally established as three novel species of *Penicillium* section *Lanata-Divaricata*. The present work highlights cultivated soils in Yunnan as a largely underexplored reservoir of soil-borne *Penicillium* diversity, and further systematic surveys across the region are likely to reveal additional undescribed *Penicillium* taxa and improve our understanding of the biogeography of this genus in southwestern China.

## Supplementary Material

XML Treatment for Penicillium longyangense

XML Treatment for Penicillium longlingense

XML Treatment for Penicillium zhenyuanense

## Additional information

### Artificial Intelligence (AI) use

The authors accept full responsibility for the content of the manuscript, including the disclosure of any use of AI.

Regarding the use of AI in the preparation of this manuscript, the authors declare the following: GPT.

**Used for**: Language, style and writing.

AI tools were used for language polishing, improvement of clarity, and preliminary brainstorming during manuscript preparation. All taxonomic decisions, morphological descriptions, phylogenetic interpretations, literature citations, references, and conclusions were independently checked, verified, and finalized by the authors, who take full responsibility for the manuscript.

### Author ORCIDs

X. Zhang https://orcid.org/0009-0003-9283-7325

S. Shi https://orcid.org/0009-0001-9721-9599

Z. Duan https://orcid.org/0009-0009-4062-089X

W. Lai https://orcid.org/0009-0001-4874-5381

Z. Yu https://orcid.org/0000-0002-6246-5861

### Data availability

All of the data that support the findings of this study are available in the main text.
